# Script of Scripts: A pragmatic workflow system for daily computational research

**DOI:** 10.1371/journal.pcbi.1006843

**Published:** 2019-02-27

**Authors:** Gao Wang, Bo Peng

**Affiliations:** 1 Department of Human Genetics, The University of Chicago, Chicago, IL, United States of America; 2 Department of Bioinformatics and Computational Biology, The University of Texas MD Anderson Cancer Center, Houston, TX, United States of America; Hebrew University of Jerusalem, ISRAEL

## Abstract

Computationally intensive disciplines such as computational biology often require use of a variety of tools implemented in different scripting languages and analysis of large data sets using high-performance computing systems. Although scientific workflow systems can powerfully organize and execute large-scale data-analysis processes, creating and maintaining such workflows usually comes with nontrivial learning curves and engineering overhead, making them cumbersome to use for everyday data exploration and prototyping. To bridge the gap between interactive analysis and workflow systems, we developed Script of Scripts (SoS), an interactive data-analysis platform and workflow system with a strong emphasis on readability, practicality, and reproducibility in daily computational research. For exploratory analysis, SoS has a multilanguage scripting format that centralizes otherwise-scattered scripts and creates dynamic reports for publication and sharing. As a workflow engine, SoS provides an intuitive syntax for creating workflows in process-oriented, outcome-oriented, and mixed styles, as well as a unified interface for executing and managing tasks on a variety of computing platforms with automatic synchronization of files among isolated file systems. As illustrated herein by real-world examples, SoS is both an interactive analysis tool and pipeline platform suitable for different stages of method development and data-analysis projects. In particular, SoS can be easily adopted in existing data analysis routines to substantially improve organization, readability, and cross-platform computation management of research projects.

This is a *PLOS Computational Biology* Software paper.

## Introduction

Computational biologists typically spend a significant portion of their time developing and using scripts written in general purpose scripting languages, such as shell, Python, R, and Ruby [1–4]. For reasons such as researchers’ inadequacy of software development skills, limited time and resources, and difficulties in and lack of need for writing portable and reusable scripts for individual projects, scripts written for daily computational research are often inadequately engineered, tested, and documented [4]. This makes it difficult for third parties, sometimes even the scripts authors, to understand and reuse scripts to reproduce prior analysis, port to different platforms, or apply to new projects.

To make scripts better suited for reproducible research, theories and implementations of scientific workflow systems [3, 5] have been introduced for script management and execution, creating what are referred to as “pipeline languages” or “pipeline tools.” Numerous pipeline tools have been developed to date [6, 7]. Most of them have rigorous interfaces and syntaxes for specification of computational tasks. They may require users to work with graphical interfaces [8], describe details of workflow steps and logics using a configuration system [9], define workflow steps as functions or classes in other scripting languages [10], or learn a new language [11–13]. Inevitably, re-factoring codes for these pipeline platforms results in notably different implementations of their non-pipeline counterparts. At an early stage of a project that involves switching between interactive analysis environments, maintenance, and synchronization of variations of the same code require a great deal of effort. Even at a late research stage, when scripts are ready to be “pipelined,” researchers may have difficulty adapting data analysis logics to comply with those of the workflow system (e.g., enforce filename patterns to connect steps of workflows) [14, 15]. Therefore, some authors argue that “pipelineitis is a nasty disease” [16]. In their view, overuse of pipelines, particularly in the early stages of projects, decreases productivity, as researchers are forced to redirect their focus from scientific problems to engineering details.

Several authors have envisioned workflow systems suitable for daily computational research. Spjuth *et*. *al*. [17] categorized workflow systems into those designed for routine processing versus those for *ad hoc* data exploration. Whereas the former should be made easy to workflow end users to use, at the cost of careful design and engineering on the developers’ end, the latter should be made easy to developers themselves, working under a framework that is “simple, lightweight, easy to install and integrate with bash and scripting languages.” Atkinson *et*. *al*. [18] argued that workflow system designs should consider researchers who have less experience in software engineering and be tailored for domain experts. In both reports, the authors called for the paramount importance to bridge the gap between interactive and batch data analysis [2], an aspect explored by only a few systems, such as YesWorkflow and NoWorkflow [19, 20], which capture workflow structure and provenance of existing scripts, but have yet to offer workflow execution features.

Motivated by the limitations of current systems for *ad hoc* data exploration, we developed Script of Scripts (SoS), a cross-platform, multilanguage scripting and workflow system designed for daily computational research. SoS features a plain-text file format for multilanguage scripting and workflow execution. It also has an optional Jupyter-based Integrated Development Environment (IDE) [21] that provides a notebook format that allows for the inclusion of scientific narratives, workflow descriptions, sample input and output, along with the embedded workflows. SoS empowers daily research applications, ranging from neatly consolidating fragments of scripts into a single executable source file for executing sophisticated workflows that harness the power of multiple remote computing environments, while keeping the entire process of script development, interactive data analysis, batch data processing, and reporting and sharing of results in a local environment familiar to users. In contrast with other workflow tools, SoS enhances existing scripts with workflow functionalities, while requiring little to no modification of the scripts themselves.

Herein we introduce SoS syntax and design, with an emphasis on exploratory analysis and prototyping features. We then describe SoS functionalities as a conventional workflow system, focusing on benefits of multiple workflow specification styles. Next, we describe the cross-platform execution and task management mechanism. Finally, we apply SoS to several well-established problems in machine learning and genomics to demonstrate how daily research tasks can be streamlined, integrated, and documented in SoS.

## Design and implementation

### SoS as a script organization tool

**Fig 1:**
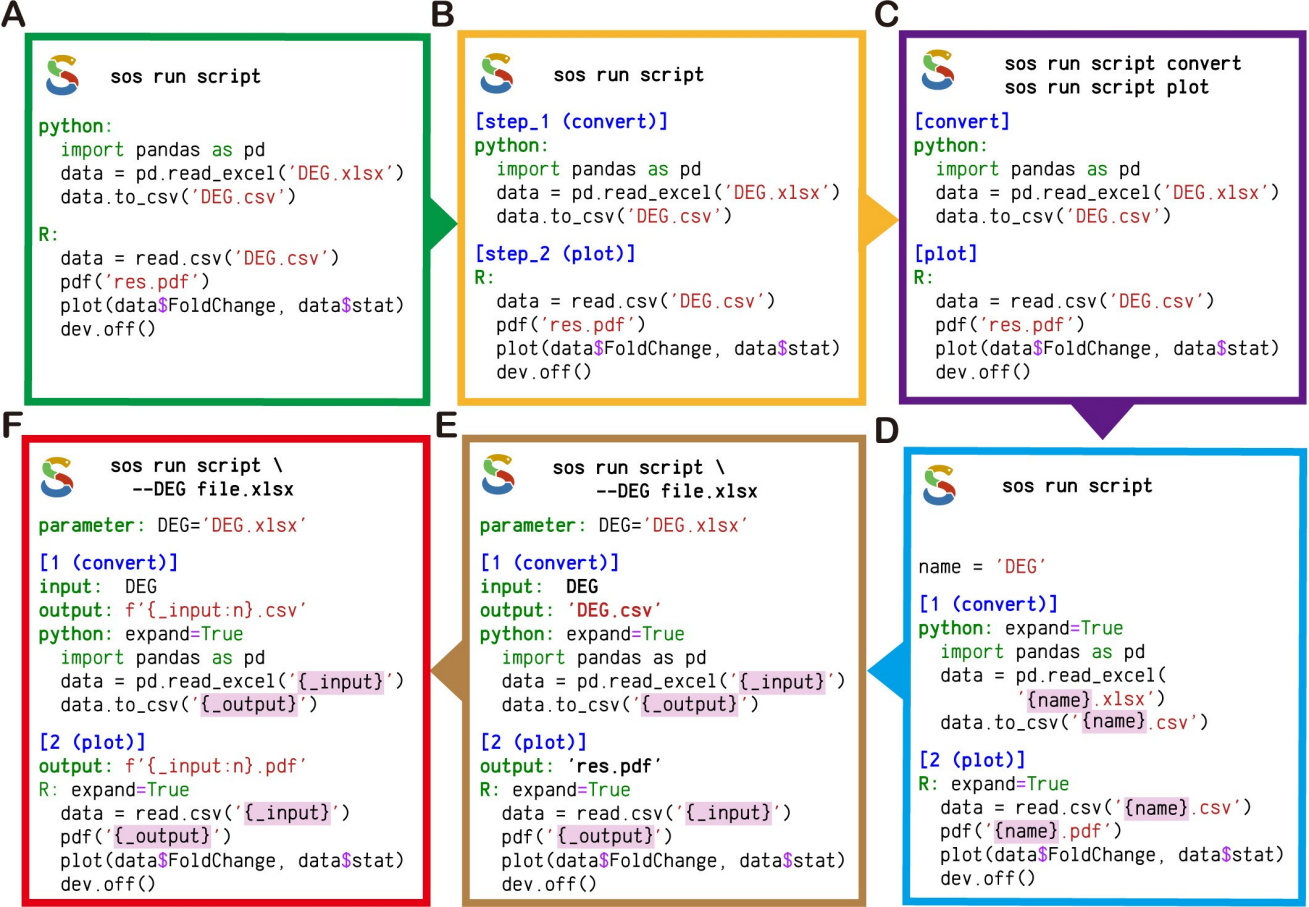
Incremental SoS scripting. (A) An SoS script integrating a Python script that converts a Microsoft Excel file to a CSV file, and an R script that plots data from the CSV file. (B) An SoS script with headers for executing two scripts as separate steps of a workflow. The section headers are numbered with optional descriptions. (C) An SoS script with headers for executing two scripts as separate workflows. (D) An SoS script that uses a global parameter and string interpolation to unify filenames. (E) An SoS script that accepts a parameter from the command line and defines input and output targets for each step. (F) An SoS script with input and output targets derived from a global parameter. SoS provides a list of format options to aid in the formatting of filenames (e.g., ‘:n’ in this example removes extensions from filenames).

Suppose data analysis can be or has been performed with a number of scripts, the scripts can be organized into one SoS script and executed by SoS. The simplest SoS scripts are merely verbatim copies of existing scripts, each with an extra word indicating the language of the script (Fig 1A). The scripts form a single-step workflow that can be executed in the order in which they are included. Optionally, “headers” can be added to code sections to separate them into steps of one or more workflows. Numerically ordered sections in SoS scripts can be executed sequentially as steps of a single workflow (Fig 1B), whereas sections with different names can be executed separately as individual workflows (Fig 1C).

If a workflow must be rerun with different options or applied to different sets of data, the scripts can be generalized to use templates, which are expanded with workflow variables using Python-formatted string literals (Fig 1D–1F). Workflow variables can be defined as parameters that accept values from the command line (Fig 1E and 1F). With the input and output of steps defined at the step level, input and output files can be accessed in the scripts as the variables “_input” and “_output” (Fig 1E and 1F). The use of SoS variables to share and pass on information, as well as the use of SoS parameters as universal lightweight argument parsers for scripts in any language, represent an easy method of consolidating scripts in different languages, while abstracting common information at the workflow level. To facilitate sharing of filenames across scripting languages, SoS implements a series of format options to render filenames in different formats (e.g., file paths, basenames, extensions, quotes for special characters) (Fig 1F).

With the option of adding comments before sections and parameter definitions, SoS enables researchers to consolidate analytic steps scattered in multiple scripts into a single, well-documented SoS script. The script can be executed using a command console. It has a proper help message (comments in the script accessed by typing command option “-h”) that summarizes available subcommands (workflow names) and parameters; it can be used to reproduce the entire data analysis or selected workflows, or it can be applied to new batches of data with different parameter settings. Even without any advanced workflow features, the ability to organize multiple scripts into a single, executable SoS script simplifies the execution of multistep, multilanguage data analysis, improving documentation, version control, and project sharing.

### Basic syntaxes of SoS workflows

With increasing data size and/or analysis complexity, “pipelinizing” a workflow for more efficient and robust executions becomes more relevant. As with many pipeline tools, rigorous workflows in SoS are characterized by steps connected with static or dynamic **input** and **output** or by target dependencies in which one step **provides** some targets that another step **depends** on. All words above in bold font are SoS keywords or options that are necessary to define complex workflows, e.g., Fig 1E and 1F.

More formally, SoS extends from Python 3.6 and allows for the use of arbitrary Python statements and modules inside an SoS workflow. An SoS script is just a Python script separated into multiple sections and interpreted by SoS. The script blocks with headers shown in Fig 1 are Python function calls written in a special script format so that the first parameter (the multiline script) can be easily specified, with or without string interpolation (option expand). Indentation of included scripts is optional, but recommended for clarity. An SoS section has a header and a body consisting of SoS directives to specify step input, output, dependent targets (if applicable), and arbitrary Python statements. The section header specifies names of and options for SoS steps and, together with the input, output, and dependent targets, determines how a workflow should be created from the sections.

The script format of function calls, section header, parameter, input, output, depends, and task (to be discussed later) statements constitute all the syntaxes that SoS adds to Python 3.6+. These statements accept options in Python syntax and control how SoS workflows and steps are executed. For example, an SoS step is usually executed once with variable _input representing all input files of the step (Fig 1). However, with input option group_by = 1, SoS divides the input files into groups of size 1 and executes the step statements (called substeps) multiple times with _input presenting each of these input groups. SoS provides multiple ways to specify input sources (e.g. from outputs of upstream steps) and to group input files, and by default executes substeps in parallel.

### Multistyle workflow system

An SoS workflow is defined as a collection of SoS steps that are connected to form a Directed Acyclic Graph (DAG). Distinct from other pipeline tools, SoS has a multistyle design to provide more than one way to construct a workflow.

The most intuitive SoS workflow style is the process-oriented style, as used with some other pipeline tools [14, 22]. In its simplest form, this style sequentially executes a series of numerically ordered steps (Fig 1, with two single-step workflows defined in Fig 1C). The step numbers do not have to be consecutive, allowing steps to be added later, if needed. Although such a workflow, by default, has a DAG with a single linear execution path, users can break the default dependencies and introduce new dependencies (edges of DAGs) by defining input, output, and dependent targets in each step. These dependent targets can be files, other SoS steps (function sos_step), outputs from upstream steps (functions output_from and named_output), software libraries, or available system resources. SoS creates a DAG from the steps and their dependencies and execute steps with met dependencies in parallel, regardless of the logical orders in which steps are specified. This process-oriented style is most naturally applied to pipelines with dynamic or multiple known outcomes. Furthermore, it is logically intuitive and thus friendly to novice users.

Another workflow style that SoS supports is the outcome-oriented or Makefile style, which relies on implicit wild-card idioms introduced with the Make utility, and adopted by some pipeline tools [15, 23]. Harnessing the power of wild-card pattern matching, the outcome-oriented style implicitly determines dependencies, and automatically builds and executes DAGs. Triggered by the specification of one or more output files through option -t of the sos run command, outcome-oriented style specification enables the flexibility of generating specific outputs by executing only the required steps and there is not necessarily a complete workflow with a final outcome. By design, it fully parallelizes the workflow engine to schedule and execute jobs. However, the logic in the outcome-oriented style is backward, which is counter-intuitive to some bioinformaticists; the conventional pattern-matched dependency specification can be tricky for steps with no obvious or dynamic outcomes. Therefore, this style is often more challenging to use and extend than process-oriented style [14].

The multistyle design of SoS alleviates the dilemma of choosing between process- and outcome-oriented styles. With SoS, researchers have the freedom to build intuitive pipelines in the process-oriented style and, as the workflow grows, convert it to the outcome-oriented style with minimal syntax changes, even though the underlying logics of the two styles are very different. In fact, many SoS users may prefer to work with the third SoS coding style: the mixed style. In this style the trunk of the workflow can be process-oriented, with dependencies in both process-oriented (depends on other steps by step names or named or unnamed outputs) and outcome-oriented (depends on pattern-matched outputs) styles. Alternatively, the trunk of the workflow can be outcome-oriented, with dependencies generated by subworkflows consisting of multiple process-oriented steps (a step depends on another workflow). The proportions of process-oriented versus outcome-oriented steps depend on the needs of specific problems. When properly applied, the mixed style usually resolves logic and syntax dilemmas encountered with the other two styles.

**Fig 2:**
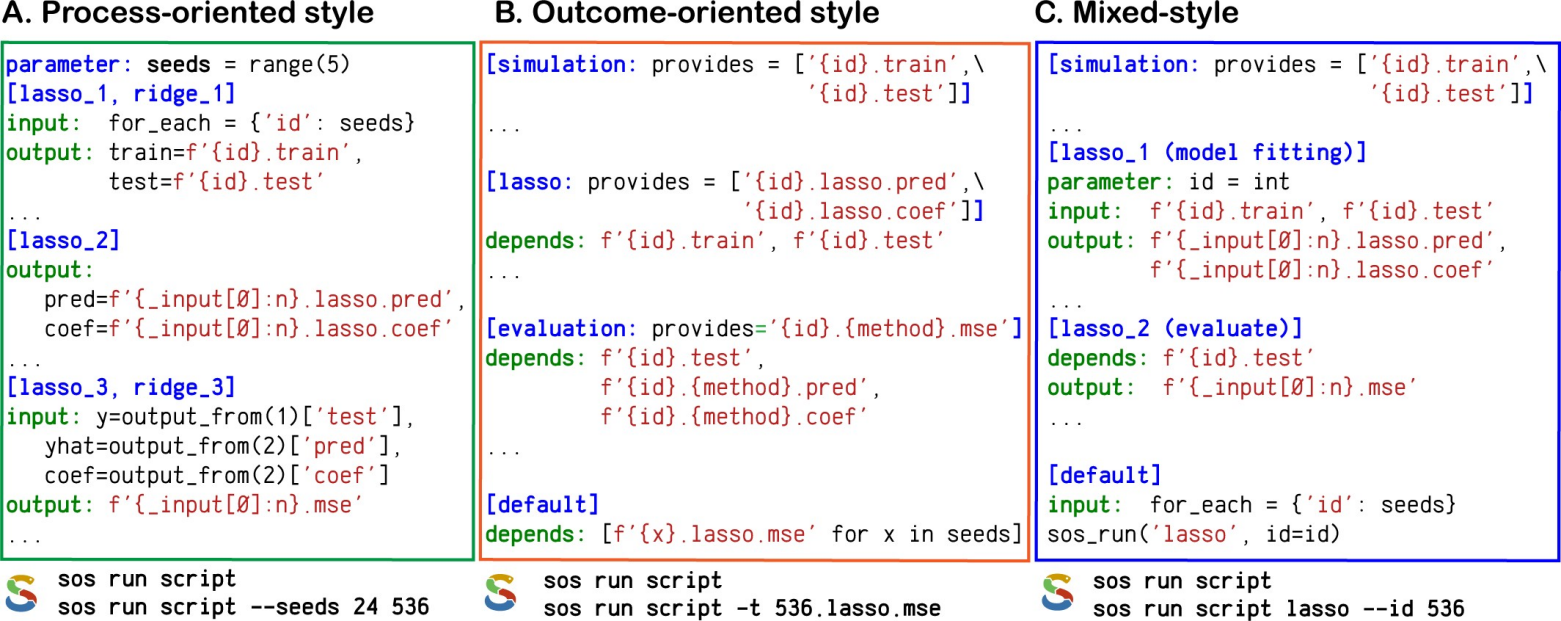
Multistyle SoS workflows. Shown are abstracted versions of implementations of high-dimensional regression workflows in SoS demonstrating the (A) process-oriented, (B) outcome-oriented, and (C) mixed workflow styles. These workflow style definitions are inspired by Leipzig [37], who classified workflow systems as either “explicit convention” (process-oriented) or “implicit convention” (outcome-oriented) frameworks. The workflow logics are primarily reflected by the SoS section names and step statements “input,” “output,” and “depends.” Full versions of these workflows are available online [28].

Fig 2 illustrates applications of the three SoS workflow styles to the same real-world problem, a simulation study elaborated on in the Results section. In this three-step numerical comparison (apart from the “default” section, which serves as a default entry point if multiple workflows are defined in the script), step 2 depends on step 1, and step 3 depends on both step 1 and step 2. As shown in Fig 2A, SoS sequentially executes one step after another, with each step analyzing all data sets in parallel. In Fig 2B, the logic is backward, as indicated by wild-card patterns, and the steps are triggered separately for each data set. In Fig 2C, the first step is “auxiliary” in the sense that it provides data to other steps only as needed; thus, users can focus on developing and expanding the core part of the simulation study (i.e., when the regression methods are executed and evaluated).

In addition to the workflows with DAGs built before execution, as described above, SoS allows for construction and execution of nested workflows within another workflow step (function sos_run in Fig 2). Combined with the ability to execute any Python statement in an SoS step, and thus execute nested workflows conditionally or repeatedly for each substep applied to subsets of input files or with different parameters (Fig 2C), SoS facilitates the creation of complex, dynamic workflows in styles suitable for the complexity of a project. Another improvement of SoS over conventional outcome-oriented workflow styles is that it extends dependency targets beyond files to prerequisites for execution of steps, such as executable commands, installed libraries, and, particularly, another SoS step or workflow. For example, dependencies can be defined by step names, in addition to step output (see the Results section for an example). From an engineering point of view, this feature addresses the known issue of file-based DAGs being impractical for large applications [14]. In practice, it further improves pipeline readability and provides support for dynamic file targets.

SoS builds a DAG from explicit and implicit step dependencies to either execute a specified workflow (process-oriented) or obtain specified targets (outcome-oriented). The DAG can be specified and resolved fully before parallel execution, or with unknown dependencies that can only be verified, created, and resolved during execution. The DAG also can be expanded dynamically with the execution of nested workflows (Fig 2C). SoS uses runtime signatures to track workflow execution (via the content of steps, environments, input, output, and dependent targets) so that successfully completed steps are ignored during the re-execution of the workflow. It also protects output files using process locks during the execution of workflows, and removes partial outputs from failed steps so that output files will only be written by one SoS instance and are saved only after successful completion of the steps. Unlike time stamps [15], step signatures in SoS are generated using checksums and are workflow-independent. Therefore, completed steps can be ignored by revised or completely different workflows, as long as the signatures stay the same. Finally, SoS allows for replacement of large intermediate files with their signatures via an operation called zap. In SoS, a “zapped” step is considered complete, unless the actual intermediate files are required in later steps in the workflow.

### Cross-platform workflow execution and job management

**Fig 3:**
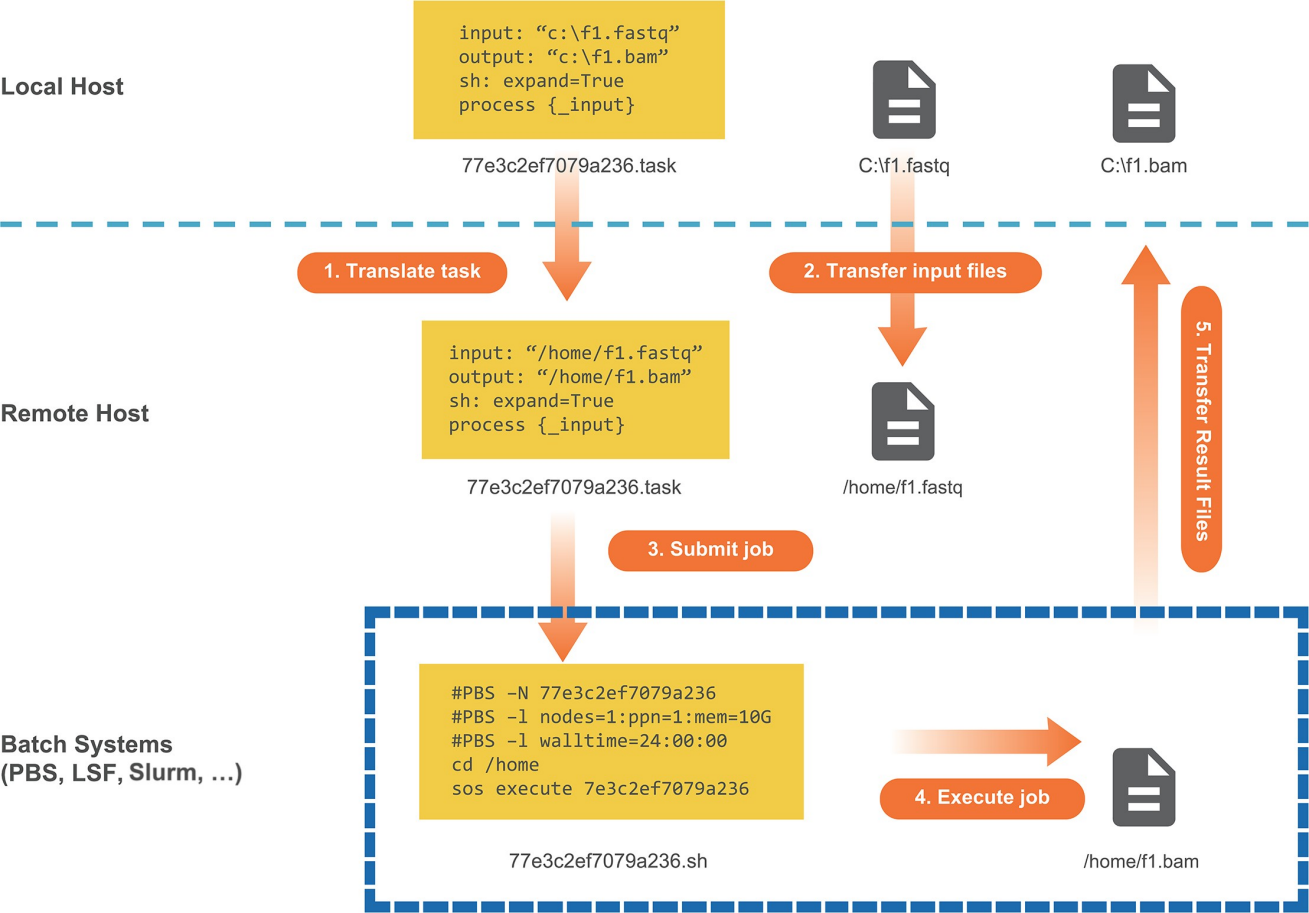
SoS task model. This diagram illustrates SoS remote task execution managed on a local machine whose file system is not shared with the remote host. 1) SoS translates the local task to a remote task via file-path mapping. 2) At the same time, SoS transfers files necessary for the computation to the remote host. 3) SoS then uses preconfigured templates to send commands to the remote job-management system to submit jobs, if applicable, or to directly execute jobs on the remote host. 4) Output files are generated at the end of computation on the remote host. 5) Results are automatically transferred back to the local host, thus completing the computational task on the local machine without direct interaction with remote hosts. Of note, different tasks can be sent to multiple remote hosts simultaneously. The local machine only provides the computational resources necessary to manage and monitor these tasks.

As research projects advance to stages involving large-scale data analysis consisting of numerous computationally intensive tasks, SoS remains particularly relevant owing to its unique cross-platform task model (Fig 3). Using a YAML-based configuration system that defines properties of all remote computing environments (hosts), SoS can submit tasks in the same workflow to one or multiple isolated hosts, such as high-performance computing clusters running various task queue systems (PBS/MOAB/LFS/Slurm), standalone workstations owned in a laboratory, and virtual machine instances hosted by cloud services. When a task is executed by different remote hosts, SoS translates paths of the task into paths on remote hosts, and optionally synchronizes input as output files as tasks are executed (Fig 3). With proper configuration of all available hosts, which can be done in advance by a system administrator for all users, running cross-platform computations involves simply sending tasks to hosts with appropriate hardware and software resources. SoS provides a series of command utilities to monitor and manage tasks. For example, sos status -q server lists the status of all tasks running on the specified server, and sos kill task_id terminates the execution of a task. These utilities unify job management on different platforms using the same interface on a local machine.

In cases when data are large and reside on remote hosts, SoS allows the execution of tasks or complete workflows on the remote host without file synchronization. For example, the input of a step can be marked as remote, so that SoS can process files that reside on a remote host, while still copying results to the local host for downstream analysis. Similarly, output files marked as remote will stay on the remote host. When both input and output files are on the remote host, it can be easier to write workflows with respect to remote paths and use option -r host to send the entire workflow to the remote host for execution. This is equivalent to, but much more convenient than copying the workflow to the remote host, logging in to that host, and executing the workflow there. The ability to analyze data remotely using local workflows simplifies the management of projects, especially when the project consists of both local and remote data analysis steps and are recorded in the same notebook.

### Container support

Whereas SoS tasks can be executed locally or distributed to multiple remote hosts, the host environment must be properly configured with required software applications. SoS addresses this problem with built-in support for docker and singularity container engines, which isolate applications and their supporting libraries and tools such that the entire tool chain and related resources are encapsulated as a bundle and can be readily executed on different platforms. By simply specifying the image and engine to use as a parameter for the script to be executed, SoS downloads the specified image, mounts appropriate directories, and executes the script inside the container. By substituting local commands and libraries with docker- or singularity-based tools [24], an SoS workflow becomes less dependent on the local runtime environment, and therefore enhances reproducibility.

## Comparison with other workflow systems

Despite vast differences in design goals, SoS remains competitive as a traditional workflow system. A unique feature of SoS is its multistyle design based on a unified dependency building scheme, which allows more flexible workflow specification and execution than uni-style workflow systems. For example, mimicking workflow systems (e.g. Galaxy [8]) that connect inputs and outputs of well-defined steps, an SoS workflow can be written as separate steps with named inputs and outputs. Because steps defined in this way can serve as both process- and outcome-oriented steps, such workflows can be used to execute any steps or generate any defined targets, and in both cases DAGs will be built dynamically from relevant steps according to the flow of data. Although such workflows lack the pattern-matching capacity of typical Makefile style workflows, they are easier to design and are especially suitable for composing data flow based workflows.

To evaluate SoS in terms of workflow features with leading bioinformatics workflow systems, we created an online table [25] that compares SoS with Snakemake [15], Nextflow [14], Galaxy [8], CWL [9] and Bpipe [22]. We acknowledge SoS’ lack of support for features such as cloud storage and distributed systems (e.g., Kubernetes), and plan to address this in future releases of SoS. The table provides details for each comparison (e.g., links to documentation), and is updated to reflect new features of workflow systems in comparison, as needed. A snapshot of the table is included with this article as S1 Table.

Among these workflow systems, comparisons between SoS and CWL are particularly interesting, because CWL was designed to be a common workflow language that SoS can potentially adopt or support. However, in contrast to CWL workflows, which requires rigorous workflow dependency instructions [26], SoS workflows are designed to be easy to read, write, and update, with a minimal amount of workflow instructions and a smooth learning curve. The workflow execution engine of SoS is designed for succinctness and flexibility, sometimes at a cost of performance (e.g. steps with unspecified input and output have to be executed sequentially). It is therefore unlikely for the SoS engine to become a favorable executor for CWL workflows compared to existing solutions.

## Results

### Multistyle workflow demonstration

We previously demonstrated the multistyle SoS workflow design with a published computational experiment of high-dimensional regression [27]. The experiment simulates labeled data sets, builds regression models, and evaluates prediction errors for Lasso and ridge regression, as outlined in Fig 2 and detailed online with links to full scripts and commands [28].

Fig 2A illustrates the process-oriented style. The default order of execution is set by the numeric order of step indices (“_1”, “_2”, “_3”). The first section, as its name “lasso_1, ridge_1” suggests, is shared step of both Lasso and ridge regression pipelines. The core algorithms “lasso_2” (and “ridge_2,” [28]), coded in different sections, use output of the first section in groups of two for training and testing data, and produce two files for parameter estimation and prediction. Outputs from first two steps both flow into “lasso_3, ridge_3,” another shared step, for performance evaluation. Dependency on both its upstream steps is explicitly configured by named inputs and outputs (function output_from), overriding the default dependency on its immediate upstream step when evaluated by the ordering of step indices. Of note, simultaneous execution of “lasso” and “ridge” have to be explicitly specified (by using one sos_run call) because dependency between the two subworkflows is ambiguous otherwise. Fig 2B illustrates the outcome-oriented style. Expected results are specified at the beginning of the script. The “default” step requires these results, which, through wild-card pattern matching, leads to the “evaluation” step, which depends on Lasso and ridge regression [28], and finally leads to execution of the “simulation” step to generate the required data set. Unlike that for process-oriented workflow style, the backward logic herein fully specifies dependencies implicitly, and is by default parallelized to execute both regression methods.

Fig 2C illustrates the mixed workflow style. It balances clarity of logic and simplicity of implementation of the computational experiment. Given the “simulation” step, or any step capable of providing training and testing data sets, this workflow is simplified to “model fitting” and “evaluation” of Lasso and ridge regression [28]. These steps are implemented in a process-oriented style, whose data dependency, similar to the outcome-oriented style, is specified by the wild-card pattern matching the “simulate” step. This style is likely more suited for this study than the other two styles, because it encourages researchers to separate data and methods in a way that the core analysis can be applied to a number of data inputs of interest.

Additionally, we demonstrate online an example “Outcome Oriented Step Targets,” a revision of Fig 2B that uses step dependencies; and an example “Mixed Style Data Flow”, a revision of Fig 2C that uses named outputs dependencies [28]. Although the execution logic remains unchanged, these alternative implementations are possibly more intuitive compared to using wild-card idiom for the problem. We also provide online a modular version of this application, which keeps regression methods implementation in separate files and execute them from both process- and outcome-oriented workflows.

### Migrating to SoS

To demonstrate migration of existing projects to SoS, we reimplemented three published bioinformatics workflows. The first workflow combines interactive analysis of differential expression, originally published as a Bioconductor tutorial but without the RNA-seq alignment workflow that we included here. The second workflow adapts a normalization and expression residual analysis pipeline for the Genotype-Tissue Expression project [29], originally written in the WDL workflow description language at the Broad institute [30]. The third workflow implements a published samtools whole genome sequencing variant calling pipeline using container tools. We demonstrate with these examples that migration to SoS requires minimal extra coding and that the codes are better organized and documented, once they are ported to SoS.

### RNA-seq alignment and differential expression analysis

The Bioconductor software project provides R libraries capable of handling aligned sequence data when performing differential expression analysis, as shown in the Bioconductor tutorial. However, external software packages and data must be used to perform the alignment to generate BAM files from FASTQ files [31, 32]. Instead of using separate fragmented scripts, our SoS implementation of the alignment pipeline is coded inside the interactive analysis notebook for differential expression analysis, which downloads all required data in SRA format from the Gene Expression Omnibus, decrypts them in FASTQ format, performs read alignment, and outputs BAM files. This alignment pipeline uses dockerized tools to guarantee reproducibility. It further benefits from the use of the Jupyter IDE to embed multipage Portable Document Format figures in interactive analysis (which precludes rendering of low-quality bitmap images for tutorial presentation in addition to high-quality vector graphics for publication), and to automatically provide an HTML-based report focusing on narratives and analysis results.

### RNA-seq normalization and expression residual analysis

This is an example of a data-preprocessing pipeline for the Genotype-Tissue Expression project, developed by the project’s analysis group [33]. Several pipelines available from this group generate gene expression count data and perform various types of preprocessing of expression and genotype data, followed by expression quantitative trait loci association mapping. Because raw RNA-seq data and genotypes are not publicly available, we focus on migrating the preprocessing pipeline to demonstrate cross-platform analysis of large data sets [34]. The original pipeline is written in WDL, which executes Python, R, and shell scripts, accompanied by separate text file documentation. We port this pipeline to SoS with few changes to the workhorse scripts, creating a single file containing all of the codes, documentation, and configurations for execution on remote machines with the following computational requirements: the normalization step requires more than 60 GB of memory, and the expression residual analysis [35] requires submitting 53 CPU-intensive batch jobs. In the SoS implementation, we use dedicated Markdown sections to emphasize computational challenges. Furthermore, with two lines of configuration (as options for the task statement), memory- and CPU-intensive jobs are sent to separate remote computers, with output automatically synchronized. The workflow is written in Jupyter IDE, which can be executed interactively or by using a command console, with an HTML report exported for circulation.

### Whole-genome sequencing mapping and variant calling pipeline

We migrated the samtools variant calling tutorial [36] to SoS workflow. All workflow steps are executed by containers we have prepared and made publicly available. Additionally, this notebook contains information on software setup instructions, steps to prepare reference genome and variants data, and examples to review intermediate output files and visualize diagnostic plots, demonstrating seamless integration of workflow steps and possible interactive exploration of step output under a unified framework.

## Discussion

Development of SoS was driven by the need for a workflow system that can be easily used for daily computational research. It is designed to 1) have a smooth learning curve, so that users with little experience with workflow systems can immediately use and benefit from it; 2) be readable, for ease of understanding and revising embedded scripts by researchers who are not familiar with SoS; 3) fit more naturally into how users already structure their analysis; and 4) allow easy access to all available computing resources with minimal extra work. Perhaps most importantly, with the assistance of the SoS Notebook and related utilities, SoS provides an integrated Jupyter-based data analysis environment that encompasses the entire spectrum of data analysis from script development, interactive data analysis, and visualization, to batch data processing and report generation. This effectively narrows the gap between interactive data analysis and batch data processing, and has the potential to become an indispensable tool for daily computational research.

We recommend and develop SoS to facilitate the verbatim inclusion of short scripts in different languages in an SoS workflow. This makes the workflow easy to read, modify, share, and reproduce. Longer scripts, especially those that are designed to be reused by multiple projects, can be saved outside of the workflow and executed by appropriate shell commands (e.g.,. use sh(‘Rscript preprocess.R’) instead of applying action R on the content of the script in SoS). However, the development of such external scripts can easily break the reproducibility of completed projects, so extra attention should be paid to version control the scripts. For scripts that are also useful to others, it is highly recommended that they be properly implemented, tested, documented, and be used in SoS as modules or libraries with stable programming interfaces. These scripts should also be versioned and made publicly available (e.g., deposited to pypi for Python modules, CRAN for R libraries, and npm for JavaScript modules) to make it easy to reproduce the workflows that depend on them.

SoS facilitates the reproducibility of bioinformatic data analysis in many ways. For the organization of data analysis, SoS helps consolidate multiple scripts into fewer SoS scripts, each with clear interfaces, and with a unified version control history for its components. For documentation, SoS generates a command-line interface with narratives of workflows and steps from the script, which facilitates keeping documentation consistent with source code. For report generation, SoS provides related functions, such as report, Rmarkdown, and pandoc to create dynamic documents that intermingle codes directly within scientific narratives in a multilanguage scripting environment. For encapsulation of the runtime environment, SoS allows for the use of containers for all scripts in the workflow. Although not the focus of this report, SoS workflows can be embedded into SoS notebooks, which can contain dynamic tables and figures, previews of output of data analysis, and Jupyter magics, such as %revisions and %sessioninfo to keep track of revisions of notebooks, session information for interpreters, and versions of libraries loaded in interactive sessions.

In conclusion, SoS is a unique workflow tool suitable for interactive data analysis and batch execution of workflows in a unified environment. With a smooth learning curve, SoS can be easily integrated into and used for all stages of computational methodology research and data-analysis projects. We believe that use of SoS in daily computational research can substantially improve the organization, readability, and cross-platform computation management, and thus reproducibility of the research.

## Availability and future directions

SoS is hosted at https://github.com/vatlab/SoS and is distributed under a 3-clause BSD license. It can be installed alone as a command line tool or as part of the SoS suite, in which an IDE and notebook interface are provided by SoS Notebook. Both classic Jupyter and JupyterLab are supported although the JupyterLab extension jupyterlab-sos is still evolving with development of JupyterLab. The SoS website (https://vatlab.github.io/sos-docs/) contains documentation, tutorials, examples of SoS, and a video library demonstrating the design and syntaxes of SoS. Although we frequently release new versions of SoS following a “release early, release often” development philosophy, we created and deposited version 0.18.1 of SoS to the Zenodo research data depository (doi: 10.5281/zenodo.1291523) for evaluation with this report. Examples described herein are available in the Publication section of the SoS documentation, as well as at Zenodo (doi:10.5281/zenodo.2537428).

## Supporting information

S1 Table Comparison of SoS with other workflow management systems. The table compare SoS with several popular bioinformatics workflow systems including Nextflow, Snakemake, Bpipe, CWL, and Galaxy, in three broad aspects: 1) basic features (syntax, file format, user interface, etc), 2) workflow features (workflow specification, dependency handling, execution and monitoring, etc), and built-in support for external tools and services (container support, HPC systems, distributed systems and cloud support). It is a snapshot of an interactive table online at https://vatlab.github.io/blog/post/comparison where comments and potential contributions from the community can be continuously incorporated through github issues or pull requests.
(XLSX)
